# Evaluation of autonomic function in children and adolescents with overactive bladder

**DOI:** 10.1590/S1677-5538.IBJU.2021.0177

**Published:** 2021-08-01

**Authors:** Rhaiana Gondim, Julia Gorjão, Ananda Nacif, Israel Franco, Ubirajara Barroso

**Affiliations:** 1 Escola Bahiana de Medicina e Saúde Pública SalvadorBA Brasil Escola Bahiana de Medicina e Saúde Pública, Salvador, BA, Brasil; 2 Yale School of Medicine Department of Urology New HavenCT United States Department of Urology, Yale School of Medicine, New Haven, CT, United States; 3 Universidade Federal da Bahia Departamento de Urologia SalvadorBA Brasil Departamento de Urologia da Universidade Federal da Bahia, Salvador, BA, Brasil

**Keywords:** Urinary Bladder, Overactive, Child, Adolescent

## Abstract

**Aims:**

To evaluate autonomic activity in children/adolescents with isolated overactive bladder.

**Materials and Methods:**

Descriptive, analytical, non-interventional, cross-sectional study conducted between February 2017 and January 2018 with individuals aged between 5 and 17 years old, with overactive bladder (OAB group) or asymptomatic (control). Neurological or anatomical abnormalities, diabetes mellitus and kidney failure constituted exclusion criteria. The DVSS and the Rome III questionnaire were applied, and heart rate variability (HRV) was assessed. The chi-square test, Student's t-test, ANOVA and the Mann Whitney U test were used in the statistical analysis.

**Results:**

41 patients with OAB and 20 controls were included. In the OAB group, there were more girls (p=0.23), more overweight/obese and constipated patients. The DVSS score was higher in the OAB group. HRV showed a higher heart rate variability at the frequency domain and LF/HF variation in the control group (p=0.02 and p=0.05 respectively). In the intergroup evaluation, LF (Hz) was predominant in the control group at the post-voiding evaluation moment (p=0.03).

**Conclusion:**

The control group demonstrated a physiological heart rate variation during the voiding process, with a predominance of sympathetic activity during urinary storage.

## INTRODUCTION

Overactive bladder (OAB) is characterized by the presence of urinary urgency, sometimes associated with daytime urinary incontinence and frequent urination. OAB could be isolated or associated with others symptoms of lower urinary tract dysfunction (LUTD) such as voiding postponement and dysfunctional voiding (1). OAB is commonly found in children and adolescents, affecting around 5-12% of children aged between 5 and 10 years old and 0.5% of adolescents up to 18 years old (2-4). Urinary tract infections and vesicoureteral reflux are often present in cases of OAB. Constipation is also commonly associated with OAB, affecting around 60% of cases and defining a condition referred as bladder and bowel dysfunction (BBD) (5).

Micturition is divided in: 1) storage phase: coordinated by the sympathetic nervous system; 2) voiding phase: coordinated by the parasympathetic nervous system. The storage phase occurs as a result of norepinephrine action on the β3 and α1 receptors, promoting relaxation of the detrusor muscle and on the α1 receptors, promoting contraction of the bladder neck. The voiding phase occurs as a result of acetylcholine action on the M3 muscarinic receptors leading to contraction of the detrusor muscle and on M2 inhibiting the effect of adenylcyclase to produce AMPc which leads to bladder relaxation. The somatic nervous system acts on the external urethral sphincter (6).

Few studies have evaluated autonomic activity in children with LUTD. Fazeli et al. reported that children with BBD (n=40) had less heart rate variability (HRV) in the frequency domain as well as less parasympathetic activity between the resting phase and bladder filling compared to the control group (7). However, this study was limited because of the heterogeneous group of patients and because only a few variables of the HRV data were analyzed. Demir et al. analyzed 40 children with OAB and 28 controls. They revealed a better urinary dynamic function at the control group compared to the OAB group (8).

The understanding of the relationship between autonomic function and urinary dynamics (urodynamics) allows us to understand more about the pathophysiology of the LUTD. The present study was designed to evaluate autonomic activity in children with isolated OAB compared to the control group.

## MATERIAL AND METHODS

This was a prospective, descriptive, analytical, non-interventional, and cross-sectional study conducted to evaluate children and adolescents aged between 5 and 17 years old attending a referral center for the diagnosis and treatment of LUTD between February 2017 and January 2018. The institution's internal review board approved the study protocol (59545316.7.0000.5544) and all of the children's legal relatives signed an informed consent form.

To be included in the OAB study group, patients had to have urinary urgency, with bell-or tower-shaped curve at uroflowmetry and no prior treatment for OAB. Patients with either neurological, cognitive or anatomical abnormalities; diagnosis of diabetes mellitus; kidney failure; hypertension or abnormal thyroid function were excluded from the study.

The urinary symptoms were evaluated using the Dysfunctional Voiding Scoring System (DVSS), validated for use in Brazilian Portuguese (9, 10). Constipation was assessed using the Rome III questionnaire. Participants with ≥2 Rome III criteria were considered constipated. Body mass index (BMI) were measured in all cases.

While the patients with urinary urgency constituted the OAB group, individuals recruited in the pediatric outpatient clinic of the same institute without urinary symptoms served as controls and were submitted to the same measurements. The criteria for admission to the control group were a DVSS score of 0 and no indication of constipation (Rome III).

All patients underwent analysis of HRV at three different moments: empty bladder (pre-voiding moment, pre VM), full bladder (full bladder moment, FBM) and after voiding (post-voiding moment, post VM). Measurements were performed using a Polar H10 heart rate monitor that transmitted data via Bluetooth. Placement of the heart rate monitor was performed by the same investigator in all participants. Heart rate data were recorded during 1 minute in three different phases: 1) After confirming that the child's bladder felt empty, he/she was asked to sit quietly for five minutes. After resting, the heart rate monitor was placed on the child's chest and the first measurement was taken (pre VM); 2) The full bladder moment (FBM) was measured when the child referred urge to void. The child was asked to rest for 5 minutes and then the data were recorded; 3) After spontaneous voiding into the uroflowmeter, the child was once again asked to sit down for five minutes before the final data recording (post VM). The collected data was sent via e-mail to another investigator, who was blinded about the study groups. The data was analyzed by Kubios 2.2 software.

Data was processed in the Time and Frequency domains with their respective endpoints being taken into consideration in the study.

1. Time domain analysis
  - Mean RR interval: changes in the intervals between successive heartbeats (RR) in milliseconds (ms);
  - Mean HR: heart rate beats (beat/minute - bpm)
  - SDNN: standard deviation of normal to normal RR intervals recorded over an interval of time, expressed in milliseconds (ms);
  - pNN50: the proportion of differences in successive RR intervals greater than 50ms;
2. Frequency domain analysis
  - a) High frequency (HF) component: variation between 0.15 to 0.4Hz that corresponds to the respiratory modulation as an indicator of parasympathetic function (vagal tone);
    - (nu): normalized units;
    - (Hz): hertz;
  - Low frequency (LF) component: variation between 0.04 and 0.15Hz that is the result of the joint action of the vagal component and the sympathetic component with sympathetic predominance.
    - (nu): normalized units;
    - (Hz): hertz;
  - LF/HF ratio: this parameter reflects absolute and relative alterations between the sympathetic and parasympathetic components. LF/HF ratio ≥1 indicates sympathetic predominance.

Data analysis was conducted using the SPSS, version 22.0 for Windows. The Kolmogorov-Smirnov test was used to verify the normality of the data distribution. To analyze the parametric variables involved in HRV, ANOVA (intragroup analysis) and Student's t-test (intergroup analysis) were used, while the Friedman test (intragroup analysis) and the Mann Whitney U test (intergroup analysis) were used for the non-parametric variables.

## RESULTS

Sixty-one patients were recruited to this study, 41 to the OAB group and 20 to the control group. In the OAB group, 23 were girls and the mean age was 9.54 years (range 5-15 years) while in the control group, 8 were girls and mean age was 9.95 years (range 6-17 years). However, there was no statistically significant difference between the groups regarding sex and age (p=0.24 and p=0.64, respectively). Analysis of BMI showed that 2 patients were underweight, 10 were of normal weigh, 9 were overweight and 9 were obese in the OAB group compared to 1 underweight, 14 of normal weight and 4 obese participants in the control group (p=0.01).

The mean DVSS score of the male's participants in the OAB group was 10.66 (95% CI: 8.96-12.37) compared to 0 (95% CI: 0-0) for the control group (p=0.008) and the female's participant in the OAB group was 11.04 (95% CI: 9.08-13.00) compared to 0 (95% CI: 0-0) for the control group (p=0.03). In the OAB group, 19 patients were classified as constipated compared to none in the control group (Table-1).

**Table 1:** Frequency distribution of constipated participants as evaluated by the Rome III criteria in the two study groups.

ROME III criteria		OAB Group	Control Group	p-value
**Straining during defecation**	0	17	15	0.005^a^
	1	4	5	
	2	2	0	
	3	6	0	
	4	12	0	
**Hard stools**	0	18	17	0.009^a^
	1	4	3	
	2	2	0	
	3	8	0	
	4	9	0	
**Sensation of incomplete evacuation**	0	26	20	0.04^a^
	1	5	0	
	2	3	0	
	3	3	0	
	4	4	0	
**Sensation of anorectal obstruction**	0	32	20	0.16^a^
	1	3	0	
	2	3	0	
	3	0	0	
	4	3	0	
**Manual maneuvers to facilitate defecation**	0	39	20	0.31^a^
	1	2	0	
	2	0	0	
	3	0	0	
	4	0	0	
**Number of evacuations (times/week)**	0-3	12	1	**p= 0.04****a**
	>4	29	19	

**OAB:** Overactive bladder.

a Chi-square analysis.

Uroflowmetry showed lower Qavg in the OAB group 6.70 (95% CI: 5.16-8.24) compared to 11.46 (95% CI: 8.02-14.89) at the control group (p=0.02) and a higher interval between the beginning to Qmax at the OAB group 8.22 (95% CI: 7.00-9.44) compared to 6.58 (95% CI 4.95-8.21) at the control group (p=0.02) (Table-2).

**Table 2:** Characteristics of uroflowmetry.

Parameters	Group		p
	OAB	Control	
Curve			0.14^a^
Bell	37	20	
Tower	4		
Urine volume	146.35 (116.47-176.22)	181.97 (130.04-233.90)	0.31^a^
Qmax	15.48 (13.00-17.97	22.67 (15.52-29.82)	0.16^a^
Qavg	6.70 (5.16-8.24)	11.46 (8.02-14.89)	0.02^a^
Interval between beginning-Qmax	8.22 (7.00-9.44)	6.58 (4.95-8.21)	0.02^a^
Duration	20.10 (17.37-19.50)	16.26 (13.02-19.50	0.39^a^

a Qui Square analysis

The intra-group analysis of time domain data, mean HR revealed a statistical difference in the control group demonstrating higher frequency at the pre VM (p=0.02). The frequency domain analysis revealed that LF (nu) had a higher score at the pre VM when compared to the FBM and post VM (p=0.01) and the LF/HF was different at the prior to voiding moment when compared to the FBM and post VM (p=0.05) in the control group. These data suggested a sympathetic activity during the pre VM as physiologically expected (Table-3).

**Table 3:** Heart rate variability within the overactive bladder (OAB) and control groups at three evaluation moments: intragroup analysis.

Parameters	Group	Evaluation Moment			p
		Pre-voiding	Full bladder	Post voiding	
Mean RR (ms)	OAB	681.7± 127.2	679.3±125.4	696.1±117.0	0.46^1^
	Control	676.7 ± 144.7	714.0 ± 160.7	699.4 ± 138.9	0.09^1^
Mean HR(bpm)	OAB	91.8 ± 16.0	92.6 ± 16.6	89.7± 14.2	0.26 1
	Control	92.7 ± 17.1	87.8 ± 16.0	89.4 ± 15.2	0.02^1^
SDNN (ms)	OAB	71.06 (38.8-89.6)	78.4 (44.3-81.6)	81.0 (42.5-83.9)	0.18 2
	Control	57.9 (32.8 -92.6)	53.9 (33.2-69.4)	72.5 (36.8-73.0)	0.84 2
pNN50 (%)	OAB	29.7(9.2-50.8)	31.5(12.7-53.3)	32.0 (13.9-50.5)	0.53 2
	Control	23.22 (2.50-41.55)	27.65 (7.72-46.75)	28.10 (5.97-46.80)	0.10^2^
LF Peak(Hz)	OAB	0.086 (0.049-0.110)	0.082 (0.043-0.116)	0.078 (0.043-0.11)	0.66^2^
	Control	0.084 (0.044-0.10)8	0.083 (0.047-0.112)	0.096 (0.054-0.147)	0.69 2
LF Power (nu)	OAB	50.56 (34.80-61.70)	51.69 (32.50-71.90)	49.04 (35.27-68.82)	0.35 2
	Control	56.45 (43.30-79.90)	43.97 (30.30-55.62)	52.48 (40.70-63.17)	0.01^2^
LF Power (ms^2^)	OAB	4093 (268-2692)	10991 (437-1858)	3148 (314-2435)	0.58 2
	Control	1579 (332-1545)	1109 (363-1195)	2788 (255-1529)	0.67 2
HF Peak(Hz)	OAB	0.252 (0.167-0.344)	0.242(0.169-0.324)	0.256 (0.170-0.336)	0.76^2^
	Control	0.253 (0.152-0.369)	0.268(0.161-0.346)	0.239 (0.152-0.358)	0.69^2^
HF Power (nu)	OAB	49.12 (38.30-65.10)	50.70 (31.10-64.10)	48.03 (27.95-67.40)	0.35 2
	Control	43.20 (38.90-56.60)	62.02 (36.40-60.92)	51.18 (34.95-65.52)	0.09 2
HF Power (ms^2^)	OAB	3760 (285-2173)	2942 (398-2418)	4197 (417-1787)	0.90 2
	Control	1593 (149-2733)	1026 (294-1568)	1566 (257-1310)	0.25 2
LF/HF ratio	OAB	1.766 (0.550-1.624)	2.276 (0.482-2.572)	1.787 (0.551-2.213)	0.35 2
	Control	2.057 (0.765-2.453)	1.612 (0.512-1.909)	1.520 (0.690-1.733)	0.05 2

1 Repeated-measures analysis of variance (ANOVA);

2 Friedman test.

Since there were more obese and constipated individuals in the OAB group an additional analysis was performed to evaluate if these characteristics constituted confounding factors. When we analyzed only eutrophic patients, we identified a statistical significance of mean RR (p=0.02) and the mean HR (p=0.002) during the intragroup analysis in the control group. When we considered only obese patients we identified higher parasympathetic activity at post VM in the control group. Likewise, when only non-constipated patients were evaluated, there were no significant changes (Supplementary Data).

The intergroup analysis evaluated separately the three different moments between the OAB and the control group. At the post VM, a statistically significant difference was found between the two groups in relation to the frequency domain (LF peak [Hz]; p=0.03) but this difference did not repeat itself at the LF Power (nu) and LF Power (ms^2^) analysis (p=0.48 and p=0.49 respectively). So, we should be conservative and not suggest a classic predominance of sympathetic activity. No statistical difference was found for any other activity (Table-4).

**Table 4:** Comparison of heart rate variability parameters between the two study groups at three different evaluation moments: intergroup analysis.

Evaluation Moment	Parameter analyzed	OAB Group (n=41)	Control Group (n=20)	p-value
Pre-voiding	Mean RR (ms)	681.7± 127.2	676.7 ± 144.7	0.87 a
	Mean HR(bpm)	91.8 ± 16.0	92.7± 17.1	0.83 a
	SDNN (ms)	70.3(39.4-88.4))	57.9 (32.8-92.6)	0.35^b^
	pNN50 (%)	29.7 (9.55-50.3)	23.2 (2.5-82.0)	0.23^b^
	LF Peak (Hz)	0.086 (0.051-0.112)	0.084 (0.044-0.108)	0.80^b^
	LF Power (nu)	50.94 (36.70-61.70)	56.45 (43.30-70.90)	0.25^b^
	LF Power (ms^2^)	3981(287-2506)	1579 (332-1545)	0.29 b
	HF Peak (Hz)	0.253 (0.168-0.343)	0.253(0.152––.369)	0.80^b^
	HF Power (nu)	48.76 (38.30-63.12)	43.20 (28.90-56.60)	0.24 b
	HF Power (ms^2^)	3653 (304-2078)	1593 (149-2733)	0.24 b
	LF/HF ratio	1.759 (0.597-2.648)	2.057 (0.765-2.453)	0.26^b^
Full bladder	Mean RR(ms)	679.3 ± 125.4	714.0 ± 160.6	0.36^a^
	Mean HR (bpm)	92.6 ± 16.6	87.8± 16.0	0.29 a
	SDNN (ms)	77.98 (45.3-81.5)	53.9 (33.2-69.4)	0.23 b
	pNN50 (%)	32.2 (14.3-54.4)	27.6 (7.72-46.7)	0.54 b
	LF Peak (Hz)	0.081 (0.043-0.113)	0.083 (0.047-0.112)	0.79^b^
	LF Power (nu)	51.32 (32.50-71.80)	43.97 (30.30-55.62)	0.25 b
	LF Power (ms^2^)	10728 (440-1857)	1109 (363-1195)	0.17 b
	HF Peak (Hz)	43.97 (30.30 -55.62)	51.18 (34.95-65.52)	0.57 b
	HF Power (nu)	43.97 (30.30 -55.62)	51.18 (34.95-65.52)	0.57 b
	HF Power (ms^2^)	2898 (413-2399)	1026 (294-1568)	0.29 b
	LF/HF ratio	2.233 (0.482-2.563)	1.611 (0.512-1.909)	0.57 b
Post-voiding	Mean RR (ms)	696.1± 117.0	699.4± 138.9	0.92^a^
	Mean HR (bpm)	89.7±14.2	89.4± 15.2	0.94^a^
	SDNN (ms)	79.71 (42.2-85.9)	72.5 (36.8-73.0)	0.31^b^
	pNN50 (%)	31.4 (13.8-48.8)	28.1 (5.97-46.8)	0.60^b^
	LF Peak (Hz)	0.075 (0.043-0.111)	0.096 (0.054-0.147)	0.03^b^
	LF Power (nu)	48.99 (35.25-69.05)	52.48 (40.70-63.17)	0.48^b^
	LF Power (ms^2^)	3049 (295-2436)	2788 (255-1529)	0.49^b^
	HF Peak (Hz)	0.256 (0.177-0.337)	0.239 (0.152-0.358)	0.37^b^
	HF Power (nu)	50.75 (30.90-64,30)	62.02 (36.40-60.92)	0.69^b^
	HF Power (ms^2^)	3955 (410-1471)	1566 (257-1310)	0,24^b^
	LF/HF ratio	1.763 (0.549-2.235)	1.520 (0.690-1.733)	0.47^b^

**OAB =** Overactive bladder.

a Student's t-test;

b Mann Whitney test

## DISCUSSION

Micturition involves a complex network of interactions between the central nervous system and the peripheral nervous system. Efforts have been made to show that autonomic alterations are capable of altering the bladder cycle of filling and emptying. The present study divided this analysis into three different physiological moments in the voiding cycle.

During the bladder-filling process, it is expected a predominance of sympathetic activity, leading to the relaxation of the muscles at the bladder outlet and contraction of the bladder neck, while during the bladder-emptying phase a predominance of parasympathetic activity is expected, promoting contraction of the detrusor muscle and leading to urethral sphincter relax.

The intragroup analysis revealed that there was a drop in heart rate on the full bladder state in the control group. Such drop in heart rate was preceded and followed by subsequent increases in heart rate. These findings would imply normal responses to bladder emptying. It suggests a bladder preparation to accommodate urine - sympathetic reflex - while the reduction in heart rate, in the moment prior to void, would indicate a predominance of parasympathetic activation preparing the bladder to empty in the control group (11, 12). Healthy individuals are expected to have a better ability to adapt to different situations and it was demonstrated by the greater oscillation in heart rate (11, 13). In the OAB group we did not see such changes, but actually noted a rise in HR at full bladder moment which would indicate a sympathetic response. Unfortunately, this did not achieve statistical significance but this rise is in accordance with what is seen in neuroimaging of patients with urgency. Patients with urgency show a marked activation of the anterior cingulate gyrus (ACG) knows as the sympathetic center in the brain. The absence of these oscillations in the OAB group could reflect autonomic dysregulation already at an early phase in bladder filling.

Analyzing the frequency domain data, the HF power increases (p=0.09) and the LF power decreases (p=0.02) at the moment prior to void which is consistent with the data in the time domain and indicates a predominance of the parasympathetic activity in the control group. In the OAB group, no changes were found in the HF or LF activity at this moment. This failure to reply the data in OAB group can be explained by a constant state of hyperactivity in the sympathetic system. It leads to a hyperstimulation of the bladder during the filling phase or leads to an inefficient communication. The miscommunication is more likely to be supported by our findings but more work needs to be done on this matter.

This study helps us elucidate and corroborate our understanding of the neurophysiology of normal voiding in controls. Our findings are in line with what would be expected from what we know about the principles of voiding. The findings in the OAB group demonstrate a different HRV pattern compared to controls and could suggest a loss of variability which would indicate a lack of signaling or more plausibly an imbalance in the sympathetic/parasympathetic equilibrium.

Patients with OAB were more likely to be constipated compared to the control group and were also more likely to be overweight or obese. These findings confirm data previously published in the literature reporting an association between the presence of urinary symptoms and the presence of obesity and abnormalities of the gastrointestinal tract (1, 5, 14, 15). The incidence of OAB is greater in obese children compared to eutrophic children. It could be explained by the frontal lobe disinhibition, resulting in alterations in eating behavior and in voiding control (15-17). About constipation, the correlation between bowel symptoms and urinary patterns, referred as BBD, is already well-established. The bowel's control is controlled by autonomic function and constipation is associated with increased sympathetic tone. Children with OAB are three times more likely to be constipated compared to children without urinary urgency (5, 18, 19). Therefore, a disruption in the sympathetic/parasympathetic balance seen in OAB can also lead to the commonly seen effect of constipation in this group of patients.

Regarding the limitations, the duration of the heart rate monitoring was too short to collect adequate VLF data. Longer records would have been more beneficial to minimize the heart rate variation. We chose the shorter time frame to reduce the risk of urinary incontinence and to decrease the patient's stress level which could be a confounding effect on the HRV data. We can assume that if we exposed the OAB group for a longer period of recording, we could expect an opposite effect with an increase in HR data and a predominance of LF activity setting a sympathetic activation. Another limitation refers to the small number of patients, both in the OAB group and in the control group; however, the sample size was similar to those used in earlier studies. For example, Fazeli et al., who evaluated 40 children with BBD and compared them to a control group of 19 children, reported less heart rate variability in patients with BBD (7). Likewise, Demir et al. evaluated 40 children with OAB and 28 controls and showed greater heart rate variability during the Valsalva maneuver in participants of the control group (8). The sample size could have affected the ability of the study to detect a difference between some variables, increasing the likelihood of a type 2 error. Finally, since there were more overweight and constipated individuals in the OAB group, the possibility cannot be ruled out that these variables could have interfered somehow in the results.

## CONCLUSIONS

The capacity for coordinated sympathetic and parasympathetic activity during the micturition process was found to be better in the control group, with a predominance of sympathetic activity during the bladder-filling phase and better heart rate variability. In patients with OAB, there is a dysregulation in the autonomic balance between the sympathetic nervous system and the parasympathetic nervous system and less sympathetic activity at the post-voiding moment.

## APPENDIX

### Supplementary Data

[Table t5]

**Table t5:** Analysis of autonomic parameters in individuals of normal weight and those without constipation in the study groups at three evaluation moments.

Parameters	Group	Evaluation moments			p-value^a^
		Pre-Voiding	Full Bladder	Post-Voiding	
		Normal Weight			
Mean RR (ms)	OAB n=9)	681.74 ± 190.10	648.18 ± 139.07	663.46 147.91	0.46^1^
	Control (n=14)	686.87 ± 155.62	739.78 ± 182.55	725.90 ± 153.78	0.02^1^
Mean HR (bpm)	OAB (n=9)	95.11± 22.07	96.63 ± 17.93	95.49 18.68	0.87^1^
	Control (n=14)	91.94 ± 18.62	85.57 ± 17.74	86.85 ± 16.73	0.002^1^
SDNN (ms)	OAB (n=9)	106.03(26.75-160.85)	55.36 (23.10-85.60)	81.11 (40.95-115.60)	0.64 2
	Control (n=14)	63.30 (33.32-101.72)	58.51 (38.80-74.20)	80.97 (40.65-72.87)	0.70^2^
pNN50 (%)	OAB (n=9)	34.77 (6.00-61.40)	24.87 (3.10-49.40)	27.03 (7.50-59.80)	0.32^2^
	Control (n=14)	26.16 (9.25-46.42)	33.09 (20.90-49.77)	33.30 (14.67-50.22)	0.27^2^
LF (nu)	OAB (n=9)	44.56 (27.50-58.50)	54.24 (44.05-67.75)	51.27 (49.10-66.50)	0.23^2^
	Control (n=14)	52.40 (35.92-67.67)	45.85 (32.10-59.80)	51.45 (37.70-66.25)	0.09^2^
LF (ms)	OAB (n=9)	11257 (145-3210)	1981 (271-3125)	1427 (231-2500)	0.45 2
	Control (n=14)	1710 (365-1638)	1338 (486-1344)	3327 (634-1451)	0.31 2
HF (nu)	OAB (n=9)	55.17 (41.20-72.15)	45.34 (32.25 -55.65)	48.44 (32.80-61.85)	0.23^2^
	Control (n=14)	47.19 (32.27-64.02)	53.73 (40.05-67.17)	69.47 (33.50-65.47)	0.19^2^
HF (ms)	OAB (n=9)	9701(211-3244)	1359 (198-2499)	2248 (214 -3197)	0.12 2
	Control (n=14)	1814 (211-3043)	1220 (549-2010)	1499 (347 – 1396)	0.45 2
LF/HF ratio	OAB (n=9)	0.952 (0.386-1.462)	1.634 (0.794-2.163)	1.279 (0.613-2.081)	0.23^2^
	Control (n=14)	1.652 (0.562-2.114)	1.210 (0.478 -1.533)	1.618 (0.611-2.036)	0.09^2^
**Obese**					
Mean RR(ms)	OAB (n=9)	670.23±132.56	703.74±166.19	735.33±147.63	0.58 1
	Control (n=4)	587.57 ± 31.29	624.20 71.39	592.12 ± 12.74	0.32^1^
Mean HR (bpm)	OAB (n=9)	93.35± 16.97	90.24± 20.76	86.02± 15.34	0.49^1^
	Control (n=4)	102.59 ± 5.22	97.40 10.91	101.73 ± 2.18	0.58^1^
SSDN (ms)	OAB (n=9)	68.81 (36.90-98.95)	66.22(47.35-91.95)	97.05 (29.85-95.45)	0.36^2^
	Control (n=4)	29.90 (23.20-35.95)	35.90 (28.75-45.65)	35.22 (27.55-46.12)	0.47^2^
pNN50 (%)	OAB (n=9)	28.02 (13.35-45.25)	33.50 (71.90-58.20)	35.86 (3.70-54.35)	0.77^2^
	Control (n=4)	2.05 (1.00-3.70)	9.52 (0.47-21.00)	9.07 (0.50-22.77)	0.42^2^
LF (nu)	OAB (n=9)	53.41 (33.40-76.55)	47.05 (19.15-69.80)	46.77 (30.40-65.40)	0.16^2^
	Control (n=4)	75.07 (65.80-86.47)	42.87 (11.69-69.32)	55.47 (52.07-59.20)	0.17^2^
LF (ms)	OAB (n=9)	3550 (408-3810)	1567 (433-1772)	6885 (319-3771)	0.23 2
	Control (n=4)	350 (173-575)	506 (167-960)	463 (196-891)	0.77 2
HF (nu)	OAB (n=9)	46.21 (23.45-66.20)	52.62 (29.95-80.90)	52.76 (34.50-68.50)	0.16 2
	Control (n=4)	24.60 (13.45-33.55)	34.37 (13.30-52.82)	43.87 (39.25-47.87)	0.36 2
HF (ms)	OAB (n=9)	3642 (238-6124)	2237 (449-3552)	11497 (308-3971)	0.71^2^
	Control (n=4)	110 (44 -182)	212 (108-413)	400 (145-840)	0.03^2^
LF/HF ratio	OAB (n=9)	1.975 (0.53-3.49)	1.617 (0.245-2.344)	3.314(0.451-1.977)	0.16^2^
	Control (n=4)	4.045 (1.967-7.367)	3.572 (0.884-7.759)	1.283 (1.090-1.519)	0.36^2^
**Constipated**					
Mean RR (ms)	OAB (n=17)	700.58 ± 142.25	694.40 ± 133.19	738.57 136.80	0.26 1
Mean HR (bpm)	OAB (n=17)	90.34 ± 17.78	91.31 ± 18.58	86.13 ± 16.56)	0.21^1^
SDNN (ms)	OAB (n=17)	93.52 (46.50-102.40)	82.17 (43.35-95.35)	112.02 (45.50-151.20)	0.49 2
pNN50 (%)	OAB (n=17)	33.71 (9.80-53.40)	35.16 (11.30-54.55)	40.15 (17.50-59.40)	0.48^2^
LF (ms)	OAB (n=17)	7856 (805-3086)	7153 (384-2475)	5512 (404-4034)	0.46^2^
LF (nu)	OAB (n=17)	51.97 (37.50-61.70)	46.62 (32.50-61.00)	48.20 (27.45-66.75)	0.16^2^
HF (ms)	OAB (n=17)	6685 (647-2626)	2273 (445-3348)	7936 (701-5333)	0.94^2^
HF (nu)	OAB (n=17)	47.70 (38.30-62.10)	53.01 (38.70-67.40)	51.57 (33.25-71.50)	0.16 2
LF/HF ratio	OAB (n=17	1.812 (0.615-1.612)	1.908 (0.483-1.580)	1.211 (0.384-2.030)	0.16 2
**Not Constipated**					
Mean RR(ms)	OAB (n=21)	657.83 ± 110.84	659.96± 118.15	659.98 95.44	0.99 1
	Control (n=20)	676.7 ± 144.7	714.0 ± 160.7	699.4 ± 138.9	0.09 1
Mean HR(bpm)	OAB (n=21)	94.15 ± 14.49	94.67 ± 14.91	93.31 12.57	0.86 1
	Control (n=20)	92.75 ± 17.18	87.85 ± 16.08	89.49 ± 15.27	0.02^1^
SDNN (ms)	OAB (n=21)	52.88 (35.45-74.45)	75.39 (45.05-76.75)	56.00 (39.65-69.75)	0.26^2^
	Control (n=20)	57.93 (32.87 -92.65)	53.93 (33.20-69.42)	72.53 (36.82-73.02)	0.84 2
pNN50 (%)	OAB (n=21)	26.51 (6.35-45.25)	28.60 (9.50-53.15)	25.45 (11.40-43.20)	0.85^2^
	Control (n=20)	23.22 (2.50-41.55)	27.65 (7.72-46.75)	28.10 (5.97-46.80)	0.10^2^
LF (ms)	OAB (n=21)	1047 (256-1393)	14098 (465-1746)	1234 (253-1429)	0.10^2^
	Control (n=20)	1579 (332-1545)	1109 (363-1195)	2788 (255-1529)	0.67^2^
LF (nu)	OAB (n=21)	49.43 (25.65-64.75)	55.80 (32.05-76.45)	49.71 (37.35-69.80)	0.26 2
	Control (n=20)	56.45 (43.30-70.90)	43.97 (30.30-55.62)	52.48 (40.70-63.17)	0.13 2
HF (ms)	OAB (n=21)	1408 (234-2001)	3484 (304-1928)	1170 (366-1316)	0.86^2^
	Control (n=20)	1593 (149-2733)	1026 (294-1568)	1566 (257-1310)	0.25^2^
HF (nu)	OAB (n=21)	50.22 (35.05-73.95)	44.00 (23.50 -67.85)	49.99 (30.05-62.10)	0.26 2
	Control (n=20)	43.20 (28.90-56.60)	51.18 (34.95-65.25)	62.02 (36.40-60.92)	0.09 2
LF/HF ratio	OAB (n=21)	1.729 (0.346-1.905)	2.573 (0.479-3.403)	2.253 (0.603-2.329)	0.26^2^
	Control (n=20)	2.057 (0.765-2.453)	1.612 (0.512-1.909)	1.520 (0.690-1.733)	0.05^2^

**OAB** = Overactive bladder;

1 Repeated-measures analysis of variance (ANOVA);

2 Friedman test.
